# Resource-Building Processes Across Life Domains: Father-Child Interactions as Starting Points for Resource Caravans

**DOI:** 10.1007/s10902-022-00523-4

**Published:** 2022-06-16

**Authors:** Petra L. Klumb, Soomi Lee, Sebastian Siegler, Bernhard Piskernik, Regina Jensen, Manuel C. Voelkle

**Affiliations:** 1 University of Fribourg, rue de Faucigny 2, 1700 Fribourg, Switzerland; 2 University of South Florida, Tampa, USA; 3 Gesundheitsförderung Schweiz, Bern, Switzerland; 4 Humboldt University Berlin, Berlin, Germany

**Keywords:** Positive father-child interactions, Perceived social resources at work, Broaden-and-build theory, Work-home resources model, Conservation-of-resources theory, Day-to-day effects

## Abstract

In two studies, we examined preconditions of resource-building processes between family and work. Focusing on positive father-child interactions, we investigated positive mood states as links between the two life domains. Fathers employed in information technology (*N*_1_ = 59) and the retail sector (*N*_2_ = 75) participated in micro-longitudinal studies, both for eight consecutive workdays. Study 1 revealed that fathers with more positive interactions with a child also reported more positive mood states and fathers with more positive mood states perceived more social resources from their supervisor during the week. The indirect effect was small but significant. In Study 2, multilevel structural-equation models did not find indirect effects at the within-person level but did show that positive father-child interactions after work were related to fathers’ positive mood states before going to bed and positive mood in the morning predicted perceived social resources from supervisors (but not from coworkers) in the forenoon. There were also positive effects of perceived social resources from supervisors on positive mood states, after work. But these did not translate into an increase in positive father-child interactions, in the evening. Hence, only single elements were supported but not the overall resource caravan.

**Supplementary Information:**

The online version contains supplementary material available at 10.1007/s10902-022-00523-4.

Most of our social interactions are positive (e.g., Campos et al., 2009) and known to affect us in beneficial ways (Maier & Klumb, 2005). For that reason, they are considered here as a starting point for resource building across life domains. While there is evidence for an affect-based link from work to home (e.g., Ilies et al., 2017), the home-to-work direction is as important but evidence for it still rare (e.g., Cho & Ciancetta, 2016 but see, e.g., Lin et al., 2020 for a motivation-based link). We integrate three theoretical approaches (conservation-of-resources theory (COR, Hobfoll, 1989), work-home resources model (W–H R model; ten Brummelhuis & Bakker, 2012), and broaden-and-build theory (Fredrickson, 2001)) and propose the home and work contexts as important passageways for positive resource caravans. Investigating this passageway, we aim to advance existing knowledge regarding the temporal dynamics of positive resource caravans between the home and work domains.

As a starting point of these resource caravans, we suggest positive parent–child interactions—often described as uplifting experiences. Positive interactions with children influence parents’ mood states and potentially their perception of social resources at work. Rather than mimicking mothers’ activities with children, fathers have a distinct influence (Lamb, 2010) and Parke (2002) describes their interactions with children as more playful, upbeat, and physically more intense and stimulating than those of mothers. Mothers and fathers also differ in their experience of parenthood, in that for the latter, parenting seems to contribute more strongly to well-being than for the former (e.g., Nelson-Coffey et al., 2019). Therefore, our approach will complement the body of research on mother–child interactions by focusing on positive interactions of fathers with their children.

To examine how positive father-child interactions influence fathers’ mood states at home as well as their perception of social resources at work we conducted two studies. We investigate between-person associations with a daily diary assessing father-child interactions and positive mood states combined with a retrospective report on perceived social resources for the whole assessment period (Study 1). To shed light on the dynamic temporal processes and the mediating role of positive mood states at the within-person level, in Study 2, we examine the within-person processes between father-child interactions, positive mood at home, perceived social resources at work, positive mood at work, and father-child interactions at home based on a micro-longitudinal design with interval-contingent sampling of experiences. In the following, we present the elements of the hypothesized resource caravans (Hobfoll et al., 2018).

## Linking Work-Home Resources Model and Broaden-and-Build Theory

As for women, combining work and family life is a source of both strain and satisfaction also for men, particularly since their family roles gained centrality (Cabrera et al., 2000). Accordingly, the W–H R model (ten Brummelhuis & Bakker, 2012) provides positive and negative perspectives on the interrelationship between work and home life and emphasizes the use and generation of resources. Resources are viewed as everything an individual perceives as helpful for goal achievement (Halbesleben et al., 2014) and they can be relatively stable or time-varying. The model distinguishes two types of resources, contextual ones situated in an individual’s social context and personal ones within the self (e.g., personality characteristics, affect quality). In either life domain, contextual resources can be the starting point of resource-gain spirals. More specifically, contextual resources in one domain (e.g., positive interactions) can help generate personal resources (e.g., mood states). Personal resources, in turn, can positively influence outcomes in the other domain (e.g., perception of and behavior towards coworkers) and thereby link life domains.

**Fig. 1:**
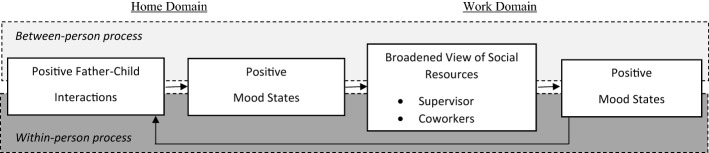
Theoretical model of the relationships between positive father-child interactions, positive mood states, perceived social resources at work, mood at work and positive father-child interactions. *Note*. All paths indicate positive relationships. See text for details

To explain resource-gain spirals, we invoke the social-functional perspective of the broaden-and-build theory of positive emotions (Fredrickson, 2001). It led us to choose the mediating personal resource, positive affective states. According to Fredrickson (2001) the experience of positive emotions is expansive and stimulates the development of additional resources. Specifically, individuals tend to have more inclusive and connected social perceptions when experiencing positive emotions. While broaden-and-build theory highlights emotions, we chose mood states as mediator between contextual resources at home and at work since mood is less fleeting and we are interested in how daily mood links two life domains. Focusing on the broadening process, we examine the role of fathers’ positive mood as a personal resource for perceiving social resources at work deemed to be a precondition of building contextual resources (e.g., actual support networks), in the longer run (ten Brummelhuis & Bakker, 2012). At the same time, we examine the role of fathers’ positive mood at the end of the workday as a personal resource for engaging in positive interactions with their child(ren), after work. Our research model combining the two theoretical models is depicted in Fig. 1. Hypothesis 1–3 refer to the between-person level while hypothesis 4–9 refer to the within-person level, i.e., to intraindividual processes over time.

## Father-Child Interactions as Contextual Resources

Our daily lives are characterized by social interactions within our role-related and personal relationships. These interactions between two or more individuals afford enjoyable activities and shared experiences and can thereby create a sense of emotional closeness and belonging (Baumeister & Leary, 1995). Moreover, they can affirm an individual’s worth (Buunk & Schaufeli 2000) and increase the subjective meaning of the performed activity (Thoits, 1983). When they involve such pleasant and meaningful experiences, social interactions are intrinsically rewarding because they satisfy the basic need for relatedness and need satisfaction, in turn, contributes to positive affect quality (Reis et al., 2000; van den Broeck et al., 2016).

Parent–child interactions are a specific case of social interactions and in the evening, parents seem to spend more time with at least one child than with the partner, exclusively (25% vs. 10% of the moments sampled, Campos et al., 2009)—a finding that emphasizes the importance of children as interaction partners. These interactions were shown to be beneficial for children’s developmental outcomes (e.g., Lamb, 2010) but knowledge regarding the effects they have on parents is still limited. Offer (2014) found that parents report feeling better in moments when they are engaged in face-to-face leisure activities with their child(ren) such as talking or playing as opposed to moments during which they are not engaged with their children. We investigate whether this momentary effect extends in time. This evidence suggests that positive father-child interactions may serve as a contextual resource that helps to generate fathers’ personal resources, specifically the energy resource ‘positive mood’, and led us to deduce the following hypothesis:

### Hypothesis 1:

Fathers who report more positive interactions with their child(ren) will experience positive mood states more frequently than fathers who report fewer positive interactions with their child.

## Positive Mood States as Personal Resources

Personal resources are viewed as a link between life domains (ten Brummelhuis & Bakker, 2012) and as antecedents of perceived job demands, resources, and behaviors (van Hooff & Taris, 2014). To explain resource-gain-spiral effects, we invoke the social-functional perspective of broaden-and-build theory (Fredrickson, 2001). The first part of this theory posits that the form of the experience of positive emotions is expansive. This broadening effect was shown by Mauss and colleagues (2011) who found that positive emotions (expressed coherently with how they are felt) are related to perceived social resources.

The second part of the theory, the build hypothesis, maintains that the ‘function of the expansive form of positive emotions is to spur the development of resources’ (Fredrickson, 2013, p. 24). Over time, when positive emotions accumulate, enduring resources should accrue via changes in the individual’s behavioral repertoire. Indeed, longitudinal studies have shown that employees who express more positive emotions (or moods) experience more social resources in their work context (from both supervisors and coworkers, Staw et al., 1994; Tsai et al., 2007) which can be the basis of an upward spiral that produces more positive emotions.

Capturing employed fathers’ experiences over a week, we propose the following:

### Hypothesis 2

Fathers with more frequent positive mood states over a week will report more social resources to be available from their supervisor at the end of the week compared to fathers with less frequent positive mood states.

Taken together, the complete resource-building process starts with positive father-child interactions which, in turn, boost fathers’ positive mood. Here, the home context provides the nurturing conditions for personal resources to develop, i.e., a resource-caravan passageway (Hobfoll et al., 2018). With the enhanced positive mood, fathers may have more inclusive social perceptions leading them to perceive more resources to be available from their supervisors. Therefore, we propose the following indirect pathway:

### Hypothesis 3

Fathers with more positive interactions with their child(ren) over a week will report more social resources at work via more frequent positive mood states.

Exclusively in Study 2, we examined whether resources obtained in the home domain would be positively associated with resources in the work domain via increased personal resources and whether resources obtained in the work domain would be positively associated with resources in the home domain via increased personal resources. To test the dynamic within-person processes along the lines of Fig. 1 we posit the following six hypotheses.

### Hypothesis 4:

On days with more positive father-child interactions (reported by the father and his spouse), fathers will experience more positive mood states at the end of day.

In addition to these immediate effects, we were interested in the effects of positive father-child interactions for fathers’ next-day positive mood states because this may have implications for the work domain (ten Brummelhuis & Bakker, 2012). Existing research showed that affective states can be transmitted from day to day (Sonnentag & Binnewies, 2013) – despite the ‘reset’ typically achieved during sleep. Moreover, an increasing number of studies show beneficial day-to-day effects of activities performed in the evening (e.g., ten Brummelhuis & Trougakos, 2013). Accordingly, we expected a positive relationship between fathers´ positive mood states in the evening and in the next morning:

### Hypothesis 5

Fathers’ positive mood states in the evening are positively related to their positive mood states in the next morning.

Regarding the broadening properties of being in a good mood stated above we expect a positive effect on perceived social connections and social interactions:

### Hypothesis 6a

On days with more positive mood states in the morning, fathers will perceive more social resources from coworkers, later that day.

### Hypothesis 6b

On days with more positive mood states in the morning, fathers will perceive more social resources from supervisors, later that day.

We then examined the reverse direction, that is, whether resources perceived in the work domain would be positively associated with resources in the home domain via increased personal resources at work.

### Hypothesis 7

On days with more perceived social resources at work, fathers will experience more positive mood states at the end of work and at the end of the day.

### Hypothesis 8

On days with more positive mood states at the end of the workday, fathers will engage in more positive father-child interactions, at home (reported by the father and his spouse).

Together, we propose the following resource caravan:

### Hypothesis 9

Fathers’ positive interactions with their child(ren) after work will be positively related to the social resources perceived the next day via more positive mood states in the evening and the next morning. These perceived resources will be related to more positive father-child interactions, in the evening, via positive mood states at the end of the workday.

## Study 1

### Materials and Methods

#### Participants

We used a sample of employees who participated in the daily diary sub-study as part of the Work, Family, and Health Study (Bray et al., 2013). Participants included full-time employees in the information technology (IT) division of a U.S. Fortune 500 company located in metropolitan areas. A total of 823 employees completed a baseline interview at the workplace and provided information on demographic and work characteristics. Of these, *N* = 222 employees with at least one child aged 9–17 living at home (i.e., target child) were eligible and invited to participate in a diary study. Of the eligible employees, 131 employees participated in an 8-day diary study and 72 of them were employed fathers. Among these, 59 fathers had at least one contact with their supervisor during the study period and provided responses on the weekly supervisor resources variable. Thus, the final sample of the first study consisted of *N*_1_ = 59 participants.

The average age of the participants was 45.49 years (*SD* = 6.36, range = 29–63). Nearly all participants (95%) were married or cohabitating. The average number of children was 2 (*SD* = 1.20, range = 1–8), and their average age was 13.20 years (*SD* = 2.37, range = 9–17). All participants were English speakers. Regarding educational levels, the vast majority (85%) had 4 years of college education or more; 14% had 1–3 years of college or technical school education; and 2% (*n* = 1) held a high-school degree, solely. The mean tenure with the company was 13 years (*SD* = 6.06, *range* = 1.5–31.92). The mean work hours were 46.68 h per week (*SD* = 5.36, *range* = 40–60) and 76% of the diary days were workdays. Most participants (78%) worked regular daytime schedule.

#### Procedure

Study 1 used deidentified data shared by the Work, Family and Health Network. The Pennsylvania State University’s Institutional Review Board (IRB) determined that this study does not involve new data collection due to the use of existing data and is exempt from further IRB review (IRB protocol number: Pro00037119). Trained interviewers obtained informed consent and then conducted computer-assisted personal interviews with the employees at the workplace at baseline. The daily diary data collection took place the month following the workplace interviews. Participants were telephoned on eight consecutive evenings and asked about their daily experiences, including interactions with target child and affect. Calls lasted about 20 min each, and participants received $150 for their diary study participation.

#### Measures

The study was administered in English. Except perceived social resources from supervisors, which were measured at the end of the diary week, all main variables were measured daily. Telephone interviewers called participants in the evening. Interview start time averaged around 7 p.m.

##### Father-Child Interactions

We used items adapted from the Parent–Child Affective Quality questionnaire (Spoth et al., 1998). The scale was designed to measure the positive and negative experiences participants may have had with the target child (aged 9–17). We chose five items that specifically asked about the frequency of positive interactions. The items read, ‘(Since this time/we spoke yesterday) how often did you (1) really listen to your child, (2) show your child that you understand how s/he feels, (3) show interest in your child’s activities, (4) make your child smile of feel happy, and (5) hug or give your child a pat on the back?’ Responses were coded as 1 = *not at all*, 2 = *once*, and 3 = *more than once*. We calculated the mean of the five items. There were only 37 days with missingness on at least one item (< 10% missing in total daily observations); these missing days were distributed across participants (e.g., 1 day or less missing within a participant on average) and thus no participant was excluded. Reliability was calculated at both the between- and within-person levels (Cranford et al., 2006) and both were adequate (between-person α = 0.99; within-person α = 0.70).

##### Positive Mood States

We used items adapted from the Positive and Negative Affect Schedule (Watson et al., 1988). Briefly, positive mood states reflect the extent to which a person feels enthusiastic, active, and alert. High positive mood is a state of high energy and pleasurable engagement. We used the mean of 10 items (enthusiastic, interested, determined, excited, inspired, alert, active, strong, proud, and attentive) to assess it. Responses on each item ranged from 1 = *none of the time* and 5 = *all of the time*. Scores were only calculated if participants answered all 10 items. Again, missingness was very small (1%) and all participants provided responses to all the items on most of the study days. Higher scores reflected more frequent positive mood states. Reliability was high at both levels (between-person α = 0.99; within-person α = 0.82).

##### Perceived Social Resources

On the last day of diary interviews, participants rated perceived support from their supervisors during the week, using the scale of Family Supportive Supervisor Behaviors (FSSB; Hammer et al., 2009). The original scale of FSSB included five items. We used two items closely related to assessing the extent of perceived support from supervisor, rather than observing specific supportive behaviors (e.g., ‘your supervisor demonstrated effective behaviors in how to juggle work and non-work issues’). The two items read, ‘In the past 8 days, (1) your supervisor made you feel comfortable talking to him/her about your conflicts between work and non-work and (2) you and your supervisor talked effectively to solve conflicts between work and non-work issues.’ Responses ranged from 1 = *strongly disagree* to 5 = *strongly agree*. The mean score was calculated, higher scores reflect greater support. The Cronbach’s alpha of the two items was 0.85.

## Analytic Strategy

Although our daily data yielded a clustered structure (59 fathers provided 472 daily observations, our outcome (i.e., weekly supervisor support) was measured only once at the end of the diary week, which motivated our focus on between-person level hypotheses. For father-child interactions and positive mood states, person means that averaged daily values across the week were created. These variables were centered at the sample means, such that higher scores reflected higher levels than others in the sample.

Hypotheses were tested using the Process Macro with bootstrapping in SAS (Hayes, 2013). The Process Macro allows for the estimation of the indirect effect, based on the product of the effect of the predictor (positive father-child interactions) on the mediator (positive mood states) and the effect of the mediator on the outcome (perceived support from supervisors). Standard errors were computed by bootstrapping, which produces confidence intervals for the indirect effects with higher statistical power and more accurate Type I error rates than Sobel test even in small samples (Hayes, 2013). In all analyses, we set the number of bootstrap samples to 10,000.

## Results

We first examined the descriptive statistics of positive father-child interactions, positive mood states, and perceived resources from supervisors reported by employed fathers (see supplementary material). On average, fathers reported that they had positive interactions with their child ‘once a day’ (*M* = 2.39, *SD* = 0.37). Fathers’ average positive mood was moderate with a mean of 2.91 (*SD* = 0.65, on a 5-point scale). The mean level of perceived resources from supervisors was close to high (*M* = 3.57, *SD* = 0.95, on a 5-point scale). Results are reported in Table 1.

**Table 1:** Parameter estimates examining the relationships between positive father-child interactions (PI), positive mood states (PM), and weekly perceived social resources from supervisors (PRS)

Hypotheses	Parameter	Estimate	*SE*	*p*-value (2-tailed)	95% CI^a^	
					LL	UL
*Direct relationships*						
1	PI → PM	1.000	0.189	< .001	0.622	1.379
	*Model fit*					
	*F* Test	27.999		< .001		
	R-squared	0.329				
2	PM → Weekly PRS	0.512	0.228	< .05	0.056	0.968
	PI → Weekly PRS	− 0.095	0.397	.813	− 0.889	0.700
	*Model fit*					
	*F* Test	3.359		< .05		
	R-squared	0.107				
*Total relationship*						
	PI → Weekly PRS	0.418	0.336	.219	− 0.255	1.091
	*Model fit*					
	*F Test*	1.544		.219		
	R-squared	0.026				
*Indirect relationships*						
3	PI → PM → PRS ^a^	0.512	0.217	< .05	0.142	1.006

*Notes. N* = 59 fathers

^a^Indirect effect remained significant after controlling for the number of children at home and the age of target child

### Father-Child Interactions and Positive Mood States

Model 1 tests the effect of positive father-child interactions on positive mood states. Fathers who reported more positive interactions with child, on average, also reported more frequent positive mood states overall across days (*B* = 1.000, *SE* = 0.189, *p* < 0.001). Thus, the data supported Hypothesis 1.

### Father-Child Interactions, Positive Mood States, and Perceived Social Resources

Model 2 tests the effects of positive mood states on weekly perceived resources from supervisors. Fathers who reported more frequent positive mood states over the week also reported perceiving greater support from their supervisors (*B* = 0.512, *SE* = 0.228, *p* < 0.05). Thus, the data supported Hypothesis 2.

As seen in the Model 4 of Table 1, there was neither a significant total association of positive father-child interactions with perceived support from supervisors (*B* = 0.418, *SE* = 0.336, *p* = 0.219) nor a direct association of positive father-child interactions with perceived support from supervisors after controlling for positive mood (*B* = −0.095, *SE* = 0.397, *p* = 0.813). But there was a significant indirect association of positive father-child interactions with perceived resources from supervisors through fathers’ positive mood states at the between-person level (*B* = 0.512, *SE* = 0.217, *p* < 0.05). Thus, Hypothesis 3 was supported, although the effects are small and should be interpreted with caution in light of the non-significant total effect of positive father-child interactions on perceived support from supervisors.

## Brief Discussion

Study 1 examined the relationships between positive father-child interactions, positive mood states, and perceived social resources from supervisors in a sample of fathers employed in the U.S. IT industry. Fathers with more positive interactions with their child reported more frequent positive mood states on average, and fathers with more frequent positive mood states also reported greater perceived support from their supervisors. These results generally support the W–H R model (ten Brummelhuis & Bakker, 2012) in that contextual resources in the home domain (i.e., positive father-child interactions) help generate personal resources (i.e., positive mood states) and personal resources influence resources in the work domain (i.e., perceived social resources). Our findings also support a part of broaden-and-build theory (Fredrickson, 2001) by showing the expansive transfer of positive mood to perceived social resources at work. For perceived social resources at work, we examined the FSSB rated by fathers given that supervisor support regarding family obligations may be specifically salient when fathers attribute their positive family life to support in the work domain (matching hypothesis, see Amstad et al., 2011, for a similar argument). Although our cross-sectional data do not allow us to test long-term accumulation of positive experiences, this study provides initial support for the broadening phenomenon of positive resources in a rarely examined context, namely daily working life and home life of employed fathers. In conclusion, we found evidence of enrichment from home to work in a U.S. sample of employed fathers.

Two major shortcomings in this study were that supervisor support was reported only at the end of the diary week and this may explain the unexpected negative effect of father-child interactions on perceived supervisor support. Moreover, only supervisors but not coworkers were included although interactions may not be the same with the two types of interaction partners (e.g., Klumb et al. 2017). Furthermore, all of our measures were based on fathers’ self-report and thus, our findings may be prone to the common-source bias (Podsakoff et al., 2003). Finally, our measure of daily positive mood states emphasized arousal (e.g., excited, enthusiastic, alert) over valence (Watson et al., 1988).

## Study 2

To cross-validate and extend the findings of Study 1, Study 2 collected data over eight consecutive workdays within a similar group of fathers working in a different industry (retail) and a different country (Switzerland) based on a micro-longitudinal design with interval-contingent sampling of experiences. Addressing some of the shortcomings of Study 1, we employed a second source of information regarding father-child interactions (spouse reports), daily measures of perceived social resources at work, as well as a measure of positive mood states that emphasizes valence rather than arousal.

## Materials and Methods

### Participants

We recruited participants in a large retail company in a French-speaking region of Switzerland. Information about the study was distributed via the human resources department (HR) and interested employees could directly contact the study coordinator so that HR involvement was restricted to the initial recruitment phase. As part of the Central European Network on Fatherhood, the study focused on fathers. Eligibility criteria were a) being male, b) working at least 50% of a full-time equivalent (i.e., 21 h per week), c) living with at least one child under 17 years (either biological or non-biological) and d) living with the family during the work week. In total, 115 fathers were interested in participating. Participation rate could not be calculated since we do not know how many employees fulfilled criteria for participation. Still, HR management judged our sample to include close to 100% of eligible employees. Of the interested employees, 31 participants did not fulfill the criteria or could not be reached despite several contact attempts. Of the 84 remaining potential participants, four withdrew before the study started. Another five participants terminated study participation in the early phase of the data collection for various reasons resulting in a sample of 75 employed fathers who completed the whole protocol (94% retention rate).

The 75 participants had an average age of 40.25 years (*Mdn* = 41, *SD* = 6.66, range = 27–54). Most participants were French speakers (72%), those speaking other languages rated their competencies as at least at a B1 level of the European Reference Framework. Almost all (73; 97%) worked between 90 and 100% of a full-time equivalent (i.e., between 38 and 42 h per week). Only two participants worked less but at least 70% (i.e., 29 h per week). The sample was highly experienced: 35 participants (46.67%) had over 20 years of experience, 31 participants (41.33%) 11–20 years, and the remaining nine participants (12%) at least 5 years. About two-thirds of participants (62.7%) had management responsibility with a mean of 31.89 employees to supervise (*Mdn* = 12, *SD* = 41.51, range = 1–150).

The diversity of the participants’ occupations was high, with more than 19 different employment functions. Most participants worked in supermarkets (55%, including sales and management), the others worked in specialized markets (9%), in the administration (8%), or other branches of the company (e.g., 5.3% in restaurants). Regarding educational levels, about one-third of these participants had completed some basic professional training (i.e., apprenticeships with some professional school, 37.5%), another third had completed advanced professional training (e.g., full-time professional school, 37.3%), 13.3% finished high school, 10.4% held a degree from a polytechnic or university, and a very small percentage did not finish obligatory schooling (1.3%).

Participants lived on average with two children in the same household (SD = 0.89, median = 2, range: 1–6). The mean age of the youngest child at home was 6.31 years (SD = 4.68 years), ranging from younger than 1 to 16 years. Seventy (93%) participants lived with their partner/spouse, and five were single fathers.

Since we were also interested in participants’ spouse’s reports on daily father-child interactions, participants asked their wives whether they would like to participate. Of the 70 partners, 65 agreed to participate in the study (participation rate of 93%). The men who dropped out and the men whose spouses did not participate were compared to the final study sample to explore potential selection effects. We found no significant differences in age, educational level, weekly work hours, or work stress (time pressure and social stressors) and strain.

The participating spouses were, on average, 38.46 years of age (*Mdn* = 39, *SD* = 6.86, range = 24–53), and 77.3% were French speakers, those who were not rated their competencies as at least at a B1 level of the European Reference Framework. In terms of education levels, the largest part of the sample had completed basic professional training (i.e., apprenticeship with some professional school, 42.4%), 22.7% held a degree from a polytechnic or university, the rest had advanced degrees of professional training (e.g., full-time professional school). More than half of the women (62.1%) had an activity level above 50% (with 21.2% employed 90–100%), while a small proportion (16.7%) were employed less than 50%. 21.2 percent were not employed at all.

Participants received 50 Swiss Francs (about 50 USD) for their participation, independently of their partner’s participation. In addition, they could take part in a lottery for a family game and ask for individual feedback primarily regarding their well-being. The local Institutional Review Board approved the study plan (protocol number 2014-140).

### Procedure

The empirical investigation consisted of three parts (first online survey, daily-assessment phase, and second online survey). After signing an informed-consent form, participants completed a survey assessing sociodemographic and personality characteristics (online or paper-and-pencil). Ahead of the daily-assessment phase, a research-team member met with each participant and provided them with an Android smartphone (*Huawei, Y300*). In a training session, the daily-assessment software *movisensXS* (version 1.0.1) was introduced, a test run carried out, and possible questions clarified.

During the following eight consecutive working days, fathers received four prompts per day which were adapted to the individual work schedules. The first questionnaire was completed in the morning, before work, and assessed mood. The second questionnaire was completed at work (noontime) and assessed mood and perceived social resources. On days when participants finished early (e.g., without a break at noon), the second questionnaire was left out resulting in a slightly smaller number of completed questionnaires for the second measurement occasion. At the end of the workday, the third measurement assessed mood either at the workplace or at home. Shortly before going to bed, the last questionnaire assessed father-child interactions and again positive mood states. Some participants worked less than eight days during the assessment period, resulting in a total of 574 working days. On days off, participants only answered the first and the last of the questionnaires. The average number of measurements per participant was 26.54 out of a maximum of 32 (*Mdn* = 27, *SD* = 4.09, range = 11–32).

Spouses completed a baseline survey and questionnaires at the end of each day their husbands participated in the study (both a paper–pencil). To provide reliable information on the exact completion time, they put the questionnaire in a postpaid envelope sealed with a timestamp of the current time and date before being sent to the research team. This information allowed us to check synchronicity of couple ratings; no questionnaires had to be excluded. The resulting number of time points per participant was 7.50 on average out of a maximum of 8 time points (*Mdn* = 8, *SD* = 0.79, range = 3–8).

### Measures

Since the study was administered in French, we used the translation–back translation procedure with two independent bilingual translators. Furthermore, we checked the understandability of the items by testing the measures with a small group of employees who did not participate in the study.

#### Father-Child Interactions

We adapted three items (out of four) previously applied by Lovejoy et al., (1999) and by Repetti and Wood (1997) and asked fathers to answer them at the end of the day. The importance of the subjective perspective notwithstanding, we asked spouses to also report father-child interactions and used those reports in addition to fathers’ self-reports as a second source of information (McAbee & Connelly, 2016). We adapted the items so that they referred to the evening and to interactions with at least one child ‘This evening … I/he played/chatted with my/our child, … my/our child and I/he laughed, … I/he was affectionate with my/our child, … I/he listened to child´s feelings and tried to understand them’**.** Items were rated on a 5-point Likert scale, ranging from 1 (*strongly disagree*) to 5 (*strongly agree*).

#### Positive Mood States

We used 3 bipolar items validated for measuring distinct core affect dimensions in daily life (Wilhelm & Schoebi, 2007). These items were developed based on the Multidimensional Mood Questionnaire (MDMQ), a German mood scale (Steyer et al., 1997). To the statement ‘At this moment I feel …’ participants responded on an eight-point scale with the endpoints 1 and 8 (both labelled with ‘*very*’). The items for positive mood states were denoted with the adjectives ‘unwell-well’, ‘good-bad’, and ‘content-discontent’ (the latter two reverse coded). We computed longitudinal reliability estimates for these affect indices based on generalizability theory, using an approach developed by Revelle and Wilt (2019; see also Cranford et al., 2006). Reliability of differences between individuals was high (0.85), reliability of within-person change from observation to observation somewhat lower (0.67) being constrained by the small number of items used for the affect index (Cranford et al., 2006).

#### Perceived Social Resources

As indicators of perceived availability of social resources, we selected two items measuring perception of available social resources (e.g., that coworkers/supervisor *can be* relied on when help is needed) out of the five items used by Frese (1999). Assuming that this perception can vary over time, we adapted these so that they assess the degree of perceived availability of resources from supervisor and coworkers in the time interval since the last measurement occasion. Participants responded to these items on a 5-point Likert scale, ranging from 1 (*strongly disagree*) to 5 (*strongly agree*).

### Analytic Strategy

The data are hierarchically structured with time points (Level 1) nested within individuals (Level 2). To account for the resulting dependencies, a multilevel structural equation model (multilevel SEM) was employed. Multilevel SEMs account for the non-independence of observations while at the same time allowing the specification of a theoretically derived SEM to test the hypothesized relationships between variables. Furthermore, they deal with measurement error by using multiple indicators for each latent variable – one of the main advantages of structural equation models (Sörbom, 1974). Our model included the latent constructs fathers’ positive mood states (PM) and positive father-child interactions (PI) in the evening, and PM in the morning of the next day before work, perceived social ressources for supervisor (PRS) and coworkers (PRC) during work, PM at the end of work, and lastly PM and PI in the evening to capture effects from work to home. Except for PI, all constructs were modeled as first-order factors, whereas PI was defined as a second-order factor with separate informant factors (father and mother) as indicators.

Following the recommendations by Enders and Tofighi (2007), we computed person-centred scores for all predictors by subtracting the person specific mean. In line with the principles of testing factorial invariance in multivariate data (Widaman et al., 1997) we constrained the measurement models to be equal for repeatedly measured variables during the day (i.e., positive mood states, positive parental interactions, supervisor and coworker resources). Specifically, we tested for strict factorial invariance by conducting Satorra-Bentler scaled chi-square difference tests. Since relaxing the constraints did not significantly improve model fit, we maintain the assumption of strict factorial invariance (i.e., invariant factor loadings, intercepts, and variances of measurement residuals). Following the same procedure, we constrained parameters of the structural model to be equivalent over time (i.e., the stability coefficients of the same variable or the regression coefficients between the same variables) while allowing for significant autocorrelations among items.

Model fit was assessed using conventional fit indices, such as chi square (${\chi }^{2}$), root-mean-square error of approximation (RMSEA), and the comparative fit index (CFI, Kline, 2015). The indirect effects were tested using PM in the evening and in the next morning as two serial mediators. There were intermittent missing values due to missed measurement occasions as well as 5 cases with completely missing spouse ratings. We used full-information-maximum-likelihood estimation to account for missing values. The model was estimated with Mplus, version 8.2 (Muthén & Muthén, 2015: 1998–2019), using robust standard errors (MLR).

## Results

We examined the relation between father-child interactions (self- and spouse-reported), positive mood states, and social resources perceived at work (for descriptive statistics see supplementary material). Fathers’ and spouses’ reports on father-child interactions converged on relatively high mean levels (self-reported: 4.07, *SD* = 0.84; spouse reported: 3.98, *SD* = 0.61) and fathers’ mood was rather positive with a mean of 6.80 (*SD* = 0.96). Fathers perceived higher levels of social resources to be available from their coworkers (2.72, *SD* = 0.82) than from their supervisors (2.01, *SD* = 0.85). Father-child interactions displayed a small positive association with positive mood states (*r* = 0.18). The intra-class correlations (ICCs) showed that there is significant between- and within-person variance in the variables measured with the daily assessments, between 34 and 52 percent of the total variance in interaction quality (self- and spouse-reported), social resources, and positive mood states was accounted for by differences between individuals.

**Fig. 2:**
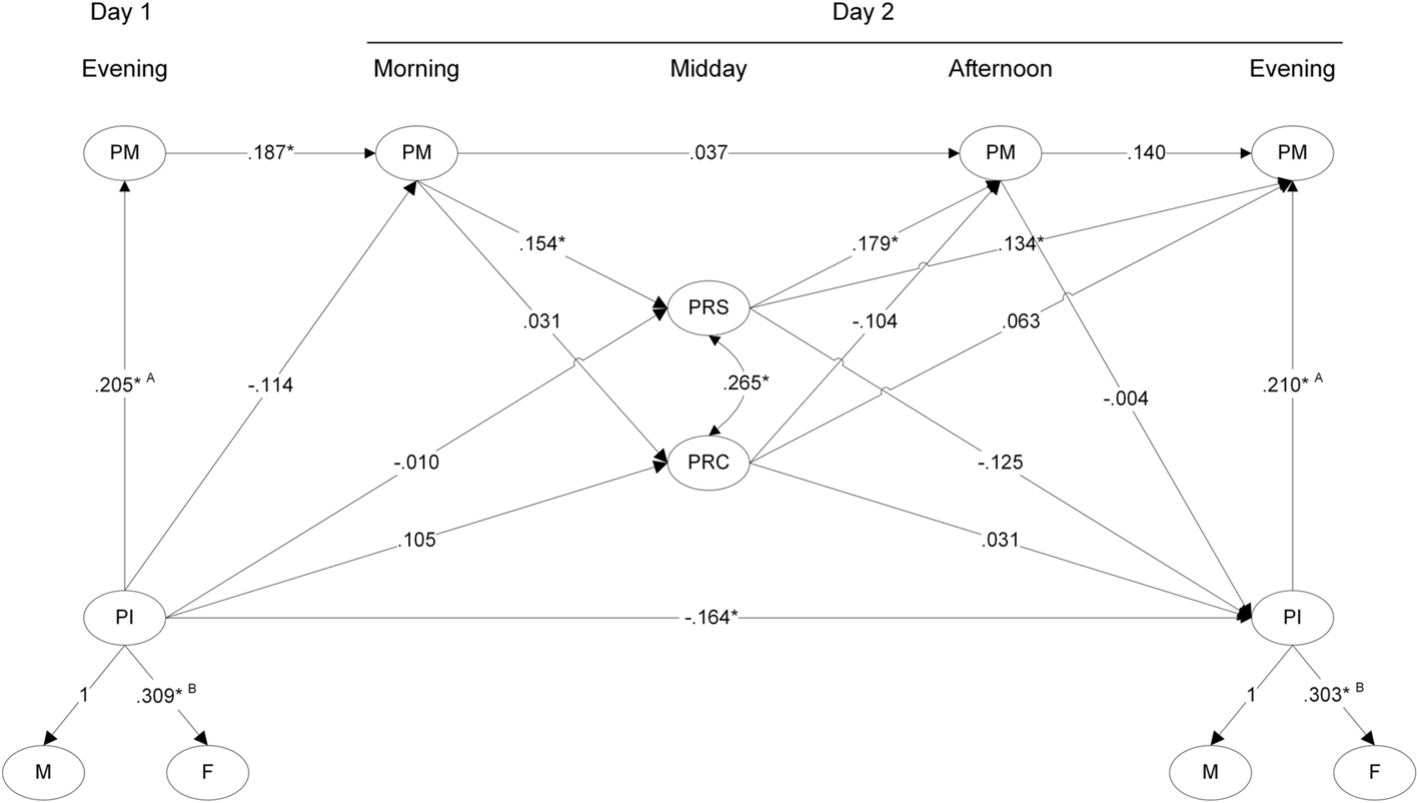
Latent part of the multilevel SEM. All paths are standardized to factor variances of one. * *p* < .05. ^A, B^ unstandardized parameters are constrained to equality. Numerical differences in Fig. 2 are due to the standardization.

The ${\chi }^{2}$-test of model fit was significant (${\chi }^{2}$(*df*) = 508.5 (382), *p*-value < 0.05), the absolute fit index *RMSEA* was 0.024, and the incremental fit index *CFI* was 0.942. Both indices represent an adequate fit of the models to the data. An overview of all structural relationships is displayed in Fig. 2. To facilitate the interpretation of the results, we report standardized coefficients, with all factor variances set to 1.

In line with our hypothesis (Hypothesis 4), above average father-child interactions were associated with higher positive mood states in the evening (first day: $\beta$ = 0.205, *p* < 0.05; second day: $\beta$ = 0.210, *p* < 0.05; note: the weights differ only due to the standardization).

Positive mood states in the evening significantly predicted next-morning affect quality ($\beta$ = 0.187, *p* < 0.05) supporting Hypothesis 5. Regarding Hypothesis 6, on days with higher positive mood states in the morning, fathers perceived more social resources to be available later in the day from supervisors ($\beta$ = 0.154, *p* < 0.05), but not from coworkers ($\beta$ = 0.031, *p* = 0.66). Hence, Hypothesis 6a was not supported whereas Hypothesis 6b was.

Regarding Hypothesis 7, on workdays during which more social resources were perceived to be available from supervisors, positive mood states were higher at the end of work ($\beta$ = 0.179, *p* < 0.05) and at the end of the day ($\beta$ = 0.134, *p* < 0.05). This was not the case, however, for those resources perceived to be available from coworkers ($\beta$ = −0.104, *p* = 0.12 and $\beta$ = 0.063, *p* = 0.34).

Hypothesis 8 regarding the influence of personal resources on contextual resources at home, and hence the direction from work to home, was not supported. There were no associations between positive mood at the end of work on positive father-child interactions in the evening (β = −0.004, *p* = 0.96). Likewise, Hypothesis 9, regarding the role of personal resources as a link between contextual resources at home and at work, was not supported. There were neither indirect effects from positive father-child interactions to social resources perceived to be available from supervisor ($\beta$ = −0.012, *p* = 0.43) or coworkers ($\beta$ = −0.002, *p* = 0.69) nor from social resources at work to father-child interactions ($\beta$= −0.001, *p* = 0.96; $\beta$ = 0.000, *p* = 0.96).

## Brief Discussion

The goal of Study 2 was to examine the effects of positive interactions between fathers and their children on the next-day perceived social resources at work via positive mood. We tested the hypothesized relationships both with self- and with spousal reports as an independent source of information.

Results supported our assumptions regarding the beneficial consequences of positive father-child interactions for fathers’ positive mood states on the same day. This result is in line with previous research on the effects of pleasurable social activities in the non-work domain (Oerlemans & Bakker, 2014) and is support for the W–H R model (ten Brummelhuis & Bakker, 2012; Sonnentag & Kruel, 2006) in that the contextual resource of pleasant interactions between fathers and their children contributes to fathers’ mood, in the evening. Future research could examine whether this effect is mediated via undoing negative arousal states and facilitating detachment from work.

Regarding the resource-building potential of positive mood states for social resources at work, results are consistent with our assumptions that employees perceive more social resources (only from supervisors, here) when they start the day in a better state. This is in line with research showing an increase in perceived social resources as consequence of accumulated positive emotions (Mauss et al., 2011) but only weak support for Fredrickson’s (2001) argumentation. Notably, while perceived availability of resources from the supervisor was lower than that perceived from coworkers, the effect of positive mood states held only for supervisor resources. It may imply that supervisors tend to be more open and supportive towards employees when employees have a positive appearance while coworkers are approachable independently of the employee’s state. At the same time, it is possible that the active initiation of an interaction with a supervisor is easier for an employee who is in a positive state, thus building the basis for perceived resources and eventually, actual support. Coworker resources, in contrast, may be particularly salient on stressful days (associated with less positive mood). In contrast to Study 1, no indirect effects (Hypothesis 9) were found in the second data set.

## General Discussion

Based on an integration of the W–H R model (ten Brummelhuis & Bakker, 2012) and COR theory (Hobfoll, 1989) with broaden-and-build theory of positive emotions (Fredrickson, 2001), we investigated effects of positive father-child interactions on perceived social resources at work via positive mood states in two studies. While there was evidence for most of the direct effects, we did not find strong support for the hypothesized resource caravans, neither on the between-person nor on the within-person level. In spite of the weak support for the hypotheses regarding the indirect effects, we can cautiously conclude that positive social interactions in the family serve as contextual resources that may be a starting point for an expansion of positive experiences (Fredrickson, 2013) and for positive resource caravans into the work domain. The conditions and consequences of engaging in an interaction with a child at a specific point in time may differ from the conditions and consequences of differences between fathers in the general level of positive interactions with their children, however.

Several explanations are possible regarding the absence of indirect effects. As the W–H R model posits, resource-building from home to work may differ as a function of fathers’ trait-like characteristics (ten Brummelhuis & Bakker, 2012), such that some fathers are more attuned to interactions with children and view their interactions in a more positive light. This may relate to obtaining personal resources (i.e., more positive mood states) and perceiving more social resources at work. Moreover, other personal energy resources such as vitality may reflect the resource-replenishing effects of social interactions more closely than positive mood states (see, e.g., Bhave & Lefter, 2017).

Although the full between-person and within-person indirect pathways (Hypotheses 3 and 9) were not or—in the first case—only weakly supported, our results provide evidence for resource transfers across domains, on both between-person and within-person levels (Hypotheses 1, 2, 4, 5, and 6). Positive father-child interactions after work were related to fathers’ momentary positive mood states at the end of the day. Fathers’ positive mood states in the morning also predicted resources perceived to be available from supervisors during the workday. These findings show that resources are shared between domains and suggest the possibility of day-to-day home-to-work enrichment (ten Brummelhuis & Bakker, 2012). Our findings shed light upon short-term processes linking positive experiences between home and work, a relatively understudied area in the work-family literature. Given that we were unable to find a full temporal process of home-to-work enrichment via positive mood states, future studies may want to identify other mediating processes. In addition, future research could also examine the links between personal resources and *actual* coworker and supervisor support or extend the view on workplace relationships beyond instrumental work-related support (see Colbert et al., 2016).

The reported resource transfers across domains have implications for occupational policies and future interventions targeting employed fathers. Given our finding that positive father-child interactions serve as a contextual resource, employers may need to devise specific strategies to not interrupt family time. This could be done, for example, by developing rules about communication after scheduled work hours. This kind of effort may become more important the more employees work remotely and the less clear the boundary between work and family life is. Further, educating supervisors on how they can better support employees’ work-home enrichment may be effective as well (Hammer et al., 2009). Finally, not all employed fathers are alike and thus discussions between supervisor and employee about individual family situations may also be helpful to achieve clear expectations that are mutually agreed upon. When organizations support employed fathers, this can affect fathers’ and family well-being, and fathers’ view of work resources and trusted social connections at work which may reduce their job strain and ultimately contribute to their family lives and performance.

## Strengths and Limitations

Our research has several strengths: First, we used daily assessments to capture short-term processes at the within-person level (cf. Beal, 2015). Second, to increase generalizability of our results, we examined the hypothesized associations in two samples. One consisted of fathers working in the North American information-technology sector, the other one of fathers working in a Swiss retail company. Third, in contrast to previous studies that mostly tested the work-to-home direction, we investigated both directions as scholars have called for. Lastly, our comprehensive analytic approach that considers between-person (Study 1) and within-person associations (Study 2) provides a more specific insight as to how positive resource caravans occur in employed fathers’ daily lives.

Finally, in Study 2 we improved methodological rigor by adding spousal-reports of father-child interactions as an additional source of information and controlling for previous values of positive mood when analyzing the effects of father-child interactions on positive mood states the next day.

Our research also has several limitations: First, we cannot rule out common-method biases due to the sole use of self-reports as outcome variables. This problem should be small, however, because we analyzed variables from multiple measurements per day, and drew on an alternative source of information, that is, spouse reports (Podsakoff et al., 2003). Second, in the Swiss sample, positive mood states were markedly higher than in the US metropolitan sample of Study 1. Apart from potential cultural differences in reporting positive mood, industry-specific differences might have played a role as retail companies often prescribe the display of specific emotions (Rafaeli & Sutton, 1989). Third, while testing our hypotheses on the time-lagged relationships between variables, we did not consider the length of time-lags between measurement occasions. Future research examining temporal relationships could test, for example, whether variables that are separated by shorter time intervals yield stronger evidence for positive resource caravans. Fourth, because of their narrow focus on (family) support our measures of perceived social resources do not reflect the whole construct. Finally, since resource gain spirals tend to be weak and take time to develop (Hobfoll et al., 2018) a longer time horizon would be needed to investigate the complete resource-building process, including manifest outcomes. This would be particularly promising via a combination of repeated daily assessments with a longitudinal design because this might explain long-term changes in personal and contextual resources with the accumulation of daily processes (see, e.g., Gerstorf et al., 2014).

## Conclusion

In the daily lives of employed fathers, we observed some evidence for positive resource caravans from home to work. Results from the two studies suggest that positive father-child interactions are important for fathers’ daily mood states and that changes in mood can contribute to a broadened view of work resources. For future research, study designs combining the examination of daily resource gains and losses with the assessment of long-term changes will provide novel insights, particularly when considering differential speeds with which the effects of gains and losses unfold.

## Supplementary Information

Below is the link to the electronic supplementary material.

Supplementary file1 (DOCX 18 kb)

Supplementary file2 (DOCX 20 kb)

Supplementary file3 (DOCX 42 kb)

## Declarations

### Data availability

The data that support the findings of this study are available from the corresponding author upon reasonable request.
