# Supplementary material for: Resource-Building Processes Across Life Domains: Father-Child Interactions as Starting Points for Resource Caravans
Source: J Happiness Stud. 2022 Jun 16;23(7):3263–83. doi: 10.1007/s10902-022-00523-4 (PMC9546948; doi:10.1007/s10902-022-00523-4)
Supplement: Supplementary file 1 — Supplementary file1 (DOCX 18 kb) [file 10902_2022_523_MOESM1_ESM.docx]

**Supplemental Table 1.** Means, standard deviations, and correlations of main variables in Study 1.

| Variable | *M* | *SD* | 1 | 2 |
| --- | --- | --- | --- | --- |
| 1. Positive father-child interactions | 2.39 | 0.37 |  |  |
| 2. Positive mood states | 2.91 | 0.65 | **.57** |  |
| 3. Perceived social resources | 3.57 | 0.95 | .16 | **.33** |

*Notes*. *N* = 59 fathers. Numbers indicate person-level data aggregated across eight diary days. Bolded correlation coefficients were significant at *p* < .05.
