# Supplementary material for: Resource-Building Processes Across Life Domains: Father-Child Interactions as Starting Points for Resource Caravans
Source: J Happiness Stud. 2022 Jun 16;23(7):3263–83. doi: 10.1007/s10902-022-00523-4 (PMC9546948; doi:10.1007/s10902-022-00523-4)
Supplement: Supplementary file 2 — Supplementary file2 (DOCX 20 kb) [file 10902_2022_523_MOESM2_ESM.docx]

**Supplemental Table 2** Means and standard deviations of all variables in Study 2

| Variable | | M | SD |
| --- | --- | --- | --- |
| Positive father-child interactions | |  |  |
|  | Indicator 1 (father) | 4.33 | 0.87 |
|  | Indicator 2 (father) | 4.1 | 1.07 |
|  | Indicator 3 (father) | 4.16 | 1 |
|  | Indicator 1 (mother) | 4.11 | 1.09 |
|  | Indicator 2 (mother) | 3.78 | 1.27 |
|  | Indicator 3 (mother) | 3.89 | 1.18 |
| Positive mood states | |  |  |
|  | Indicator 1 (T1) | 6.61 | 1.29 |
|  | Indicator 2 (T1) | 6.75 | 1.29 |
|  | Indicator 3 (T1) | 6.38 | 1.42 |
|  | Indicator 1 (T3) | 6.54 | 1.29 |
|  | Indicator 2 (T3) | 6.58 | 1.37 |
|  | Indicator 3 (T3) | 6.51 | 1.41 |
|  | Indicator 1 (T4) | 6.96 | 1.15 |
|  | Indicator 2 (T4) | 6.83 | 1.23 |
|  | Indicator 3 (T4) | 6.61 | 1.42 |
| Perceived social resources | |  |  |
|  | Indicator 1 (coworkers) | 2.97 | 1.37 |
|  | Indicator 2 (coworkers) | 2.64 | 1.41 |
|  | Indicator 1 (supervisor) | 2.23 | 1.45 |
|  | Indicator 2 (supervisor) | 2.26 | 1.48 |
