# Supplementary material for: Resource-Building Processes Across Life Domains: Father-Child Interactions as Starting Points for Resource Caravans
Source: J Happiness Stud. 2022 Jun 16;23(7):3263–83. doi: 10.1007/s10902-022-00523-4 (PMC9546948; doi:10.1007/s10902-022-00523-4)
Supplement: Supplementary file 3 — Supplementary file3 (DOCX 42 kb) [file 10902_2022_523_MOESM3_ESM.docx]

**Supplemental Table 3.**  Inter-variable correlation matrix of all variables in Study 2

|  |  |  |  | Positive father-child interactions | | | | | | | | | | | | | | |  | Positive mood states | | | | | | | | | | | | | | |  | Social resources | | | | |
| --- | --- | --- | --- | --- | --- | --- | --- | --- | --- | --- | --- | --- | --- | --- | --- | --- | --- | --- | --- | --- | --- | --- | --- | --- | --- | --- | --- | --- | --- | --- | --- | --- | --- | --- | --- | --- | --- | --- | --- | --- |
|  |  |  |  | Father | | | | | | |  | Mother | | | | | | |  | Day 1 | | |  | Day 2 | | | | | | | | | | |  |  |  |  |  |  |
|  |  |  |  | Day 1 | | |  | Day 2 | | |  | Day 1 | | |  | Day 2 | | |  | Evening | | |  | Morning | | |  | Afternoon | | |  | Evening | | |  | Supervisor | |  | Coworkers | |
|  |  |  |  | I1 | I2 | I3 |  | I1 | I2 | I3 |  | I1 | I2 | I3 |  | I1 | I2 | I3 |  | I1 | I2 | I3 |  | I1 | I2 | I3 |  | I1 | I2 | I3 |  | I1 | I2 | I3 |  | I1 | I2 |  | I1 | I2 |
| Positive father-child interactions | Father | Day 1 | I1 |  | .58 | .50 |  | .28 | .34 | .30 |  | .19 | .20 | .19 |  | .08 | .05 | .06 |  | .22 | .14 | .09 |  | .16 | .15 | .02 |  | .16 | .12 | .09 |  | .10 | .06 | .02 |  | .01 | -.01 |  | -.03 | -.05 |
|  |  |  | I2 | .58 |  | .68 |  | .29 | .51 | .41 |  | .27 | .35 | .32 |  | .11 | .13 | .14 |  | .14 | .11 | .09 |  | .13 | .14 | .09 |  | .14 | .09 | .05 |  | .04 | .03 | .07 |  | .07 | .03 |  | .01 | .06 |
|  |  |  | I3 | .50 | .68 |  |  | .28 | .45 | .46 |  | .24 | .27 | .28 |  | .14 | .16 | .17 |  | .16 | .17 | .09 |  | .15 | .17 | .06 |  | .22 | .15 | .12 |  | .17 | .17 | .15 |  | .01 | .02 |  | -.04 | .05 |
|  |  | Day 2 | I1 | .28 | .29 | .28 |  |  | .56 | .51 |  | .25 | .24 | .11 |  | .16 | .14 | .16 |  | .04 | .11 | .05 |  | .09 | .13 | .10 |  | .12 | .03 | .01 |  | .24 | .17 | .10 |  | -.01 | -.01 |  | .05 | -.01 |
|  |  |  | I2 | .34 | .51 | .45 |  | .56 |  | .69 |  | .24 | .25 | .17 |  | .24 | .32 | .30 |  | .00 | .03 | .04 |  | .09 | .16 | .16 |  | .11 | .06 | .02 |  | .14 | .14 | .10 |  | .04 | -.03 |  | .04 | .05 |
|  |  |  | I3 | .30 | .41 | .46 |  | .51 | .69 |  |  | .26 | .25 | .15 |  | .23 | .25 | .26 |  | .05 | .12 | .04 |  | .14 | .17 | .14 |  | .13 | .11 | .00 |  | .19 | .19 | .12 |  | .00 | .02 |  | -.08 | .02 |
|  | Mother | Day 1 | I1 | .19 | .27 | .24 |  | .25 | .24 | .26 |  |  | .64 | .58 |  | .27 | .24 | .23 |  | .07 | .08 | .11 |  | .07 | .00 | .08 |  | .04 | .02 | .02 |  | .05 | .02 | .02 |  | .03 | .09 |  | -.02 | -.01 |
|  |  |  | I2 | .2 | .35 | .27 |  | .24 | .25 | .25 |  | .64 |  | .71 |  | .24 | .35 | .23 |  | .09 | .09 | .09 |  | .09 | .02 | .03 |  | .09 | .10 | .07 |  | .10 | .06 | .05 |  | .06 | .16 |  | .11 | .09 |
|  |  |  | I3 | .19 | .32 | .28 |  | .11 | .17 | .15 |  | .58 | .71 |  |  | .20 | .22 | .23 |  | .11 | .13 | .14 |  | .05 | -.02 | .01 |  | .11 | .08 | .00 |  | .06 | -.02 | .00 |  | .09 | .15 |  | .03 | .08 |
|  |  | Day 2 | I1 | .08 | .11 | .14 |  | .16 | .24 | .23 |  | .27 | .24 | .20 |  |  | .68 | .59 |  | .02 | .04 | -.09 |  | .11 | .01 | -.05 |  | .03 | -.03 | .02 |  | .09 | .11 | .13 |  | .00 | .02 |  | -.04 | -.05 |
|  |  |  | I2 | .05 | .13 | .16 |  | .14 | .32 | .25 |  | .24 | .35 | .22 |  | .68 |  | .71 |  | .04 | .06 | .00 |  | .10 | .05 | .01 |  | .06 | .00 | .06 |  | .07 | .10 | .13 |  | .01 | .06 |  | .07 | .02 |
|  |  |  | I3 | .06 | .14 | .17 |  | .16 | .30 | .26 |  | .23 | .23 | .23 |  | .59 | .71 |  |  | .07 | .07 | -.01 |  | .10 | .04 | .00 |  | .10 | .03 | .03 |  | .13 | .15 | .19 |  | .05 | .10 |  | .05 | .02 |
| Positive mood states | Day 1 | Evening | I1 | .22 | .14 | .16 |  | .04 | .00 | .05 |  | .07 | .09 | .11 |  | .02 | .04 | .07 |  |  | .63 | .52 |  | .41 | .34 | .29 |  | .53 | .44 | .41 |  | .47 | .40 | .37 |  | -.01 | -.02 |  | -.06 | -.05 |
|  |  |  | I2 | .14 | .11 | .17 |  | .11 | .03 | .12 |  | .08 | .09 | .13 |  | .04 | .06 | .07 |  | .63 |  | .66 |  | .41 | .52 | .40 |  | .40 | .49 | .45 |  | .38 | .47 | .40 |  | .03 | .05 |  | -.02 | .03 |
|  |  |  | I3 | .09 | .09 | .09 |  | .05 | .04 | .04 |  | .11 | .09 | .14 |  | -.09 | .00 | -.01 |  | .52 | .66 |  |  | .33 | .41 | .44 |  | .36 | .39 | .49 |  | .36 | .33 | .40 |  | -.01 | -.03 |  | -.08 | .05 |
|  | Day 2 | Midday | I1 | .16 | .13 | .15 |  | .09 | .09 | .14 |  | .07 | .09 | .05 |  | .11 | .10 | .10 |  | .41 | .41 | .33 |  |  | .62 | .5 |  | .49 | .45 | .38 |  | .43 | .39 | .31 |  | .00 | .08 |  | .01 | .05 |
|  |  |  | I2 | .15 | .14 | .17 |  | .13 | .16 | .17 |  | .00 | .02 | -.02 |  | .01 | .05 | .04 |  | .34 | .52 | .41 |  | .62 |  | .67 |  | .41 | .50 | .52 |  | .35 | .50 | .45 |  | .06 | .05 |  | .05 | -.01 |
|  |  |  | I3 | .02 | .09 | .06 |  | .10 | .16 | .14 |  | .08 | .03 | .01 |  | -.05 | .01 | .00 |  | .29 | .40 | .44 |  | .50 | .67 |  |  | .37 | .44 | .47 |  | .31 | .41 | .48 |  | .08 | .08 |  | .11 | .14 |
|  |  | Afternoon | I1 | .16 | .14 | .22 |  | .12 | .11 | .13 |  | .04 | .09 | .11 |  | .03 | .06 | .10 |  | .53 | .40 | .36 |  | .49 | .41 | .37 |  |  | .67 | .54 |  | .53 | .45 | .40 |  | .00 | .13 |  | .01 | .10 |
|  |  |  | I2 | .12 | .09 | .15 |  | .03 | .06 | .11 |  | .02 | .10 | .08 |  | -.03 | .00 | .03 |  | .44 | .49 | .39 |  | .45 | .50 | .44 |  | .67 |  | .67 |  | .46 | .51 | .45 |  | .03 | .10 |  | .04 | .05 |
|  |  |  | I3 | .09 | .05 | .12 |  | .01 | .02 | .00 |  | .02 | .07 | .00 |  | .02 | .06 | .03 |  | .41 | .45 | .49 |  | .38 | .52 | .47 |  | .54 | .67 |  |  | .41 | .39 | .50 |  | -.07 | .00 |  | .05 | .03 |
|  |  | Evening | I1 | .10 | .04 | .17 |  | .24 | .14 | .19 |  | .05 | .10 | .06 |  | .09 | .07 | .13 |  | .47 | .38 | .36 |  | .43 | .35 | .31 |  | .53 | .46 | .41 |  |  | .61 | .52 |  | .02 | .02 |  | .00 | -.01 |
|  |  |  | I2 | .06 | .03 | .17 |  | .17 | .14 | .19 |  | .02 | .06 | -.02 |  | .11 | .10 | .15 |  | .40 | .47 | .33 |  | .39 | .50 | .41 |  | .45 | .51 | .39 |  | .61 |  | .67 |  | .03 | .04 |  | .07 | .03 |
|  |  |  | I3 | .02 | .07 | .15 |  | .10 | .10 | .12 |  | .02 | .05 | .00 |  | .13 | .13 | .19 |  | .37 | .40 | .40 |  | .31 | .45 | .48 |  | .40 | .45 | .50 |  | .52 | .67 |  |  | -.01 | .03 |  | .06 | .08 |
| Social resources | Super-  visor | | I1 | .01 | .07 | .01 |  | -.01 | .04 | .00 |  | .03 | .06 | .09 |  | .00 | .01 | .05 |  | -.01 | .03 | -.01 |  | .00 | .06 | .08 |  | .00 | .03 | -.07 |  | .02 | .03 | -.01 |  |  | .73 |  | .28 | .16 |
|  |  |  | I2 | -.01 | .03 | .02 |  | -.01 | -.03 | .02 |  | .09 | .16 | .15 |  | .02 | .06 | .10 |  | -.02 | .05 | -.03 |  | .08 | .05 | .08 |  | .13 | .10 | .00 |  | .02 | .04 | .03 |  | .73 |  |  | .28 | .40 |
|  | Co-  workers | | I1 | -.03 | .01 | -.04 |  | .05 | .04 | -.08 |  | -.02 | .11 | .03 |  | -.04 | .07 | .05 |  | -.06 | -.02 | -.08 |  | .01 | .05 | .11 |  | .01 | .04 | .05 |  | .00 | .07 | .06 |  | .28 | .28 |  |  | .51 |
|  |  |  | I2 | -.05 | .06 | .05 |  | -.01 | .05 | .02 |  | -.01 | .09 | .08 |  | -.05 | .02 | .02 |  | -.05 | .03 | .05 |  | .05 | -.01 | .14 |  | .10 | .05 | .03 |  | -.01 | .03 | .08 |  | .16 | .40 |  | .51 |  |
